# Conjunctival Microbiota in Patients With Type 2 Diabetes Mellitus and Influences of Perioperative Use of Topical Levofloxacin in Ocular Surgery

**DOI:** 10.3389/fmed.2021.605639

**Published:** 2021-04-06

**Authors:** Xiangjia Zhu, Ling Wei, Xianfang Rong, Yinglei Zhang, Qian Zhang, Xiaofeng Wen, Wenwen He, Keke Zhang, Feng Chen, Lai Wei, Yi Lu

**Affiliations:** ^1^Department of Ophthalmology and Eye Institute, Ear, Nose and Throat (ENT) Hospital of Fudan University, Shanghai, China; ^2^National Health Commission (NHC) Key Laboratory of Myopia (Fudan University), Key Laboratory of Myopia, Chinese Academy of Medical Science, Shanghai, China; ^3^Shanghai Key Laboratory of Visual Impairment and Restoration, Shanghai, China; ^4^Central Laboratory, Peking University School and Hospital of Stomatology, Beijing, China; ^5^State Key Laboratory of Ophthalmology, Zhongshan Ophthalmic Center, Sun Yat-sen University, Guangzhou, China

**Keywords:** type 2 diabetes mellitus, conjunctival microbiota, levofloxacin, cataract surgery, *Staphylococcus*

## Abstract

**Background:** Patients with type 2 diabetes mellitus (T2DM) are prone to ocular surface infections. We therefore characterized the conjunctival microbiome of T2DM patients and the influence of topical levofloxacin to investigate whether a dysbiosis is associated with this phenomenon.

**Methods:** Conjunctival microbiome of 79 T2DM patients and 113 non-diabetic controls was profiled using the 16S rDNA sequencing approach. Furthermore, 21 T2DM and 14 non-diabetic patients who underwent cataract surgeries were followed up perioperatively and the influence of pre- and post-operative levofloxacin on the conjunctival microbiome was further investigated prospectively and compared longitudinally.

**Results:** The α-diversity of the conjunctival microbiota was significantly higher in T2DM patients than in controls (*P* < 0.05). Significant differences in both composition and function of the conjunctival microbiome were identified on the ocular surface of T2DM patients as compared to non-diabetic controls. Particularly, phylum *Bacteroidetes* and *Fusobacteria*, genus *Pseudomonas, Haemophilus*, and *Empedobacter* were enriched, while genus *Streptococcus* was reduced on the T2DM ocular surface. Microbial genes functioning of bacterial chemotaxis was elevated in the conjunctival microbiome of T2DM patients. Furthermore, compared to the initial status, several genera including *Staphylococcus* were more abundant in the conjunctival microbiome of T2DM patients after 3-days use of preoperative levofloxacin topically, while no genus was more abundant in the non-diabetic follow-up group. No difference was observed between initial status and 7 days after ceasing all postoperative medications in both diabetic and non-diabetic follow-up groups.

**Conclusions:** The conjunctival microbiome of T2DM patients was more complex and may respond differently to topical antibiotics.

## Introduction

It's estimated 700 million people may suffer from diabetes mellitus by the year 2045 (1). Type 2 diabetes mellitus (T2DM) might make up about 90% of the cases (2), resulting in a large burden of the world. Diabetic patients are known to be vulnerable to infections throughout the whole body (3), with higher positive rates of bacterial isolation from various tissues (4–6). They are also prone to the ocular infections in lids, conjunctiva, and cornea (7, 8), as well as more complications after ocular surgeries (9), leading to worse visual prognosis (10, 11).

Normally, the ocular surface is directly exposed to the environment and plays an essential role in the surface defense system (12, 13). Many ocular infections are closely related to the dysbiosis of ocular surface. *Staphylococcus aureus*, Coagulase negative *Staphylococci, Streptococcus pneumoniae*, and *Pseudomonas aeruginosa* are the leading isolates in ocular infections (14). Yet previous researches on ocular surface flora which relied on isolation and culturing methods could only reveal limited culturable microbes. As the development of next-generation sequencing (NGS) technology, studies have shifted to evaluate the composition and functions of microbiome at a genus level, which is also time-saving and high-throughput.

The composition of ocular surface microbiome in healthy subjects has been well-illustrated previously. There were only a few studies focused on the microbial characteristics of diabetic patients with 16S rDNA sequencing technique, either with small sample size (15, 16), or the age and sex were not compatible (16). On the other hand, levofloxacin, a third generation of fluoroquinolone drug, is currently the most widely-used anti-infective and perioperative antibiotics with a broad spectrum (more tend to against Gram-negative bacteria) (17). However, in diabetic patients, it remained unclear whether the conjunctival microbiome had a different response to its topical use.

Therefore, in order to investigate whether the dysbiosis of ocular surface is associated with higher risks of ocular infections in T2DM patients, we characterized the conjunctival microbiome of patients with T2DM, and investigated the influence of perioperative use of levofloxacin on the conjunctival microbiome.

## Methods

This study was approved by the Ethics Committee of Eye and Ear, Nose, and Throat Hospital of Fudan University (No. 2013021). All procedures adhered to the tenets of the Declaration of Helsinki, and signed informed consents were obtained from all participants.

### Subjects

Totally 80 T2DM patients and 150 non-diabetic controls were consecutively recruited from January to June in 2019. The inclusion criteria were T2DM patients with a controlled and stable blood glucose (fasting blood-glucose <8.3 mmol/L or HbA1c level ≤ 7%), and free of systemic administration or topical use of eyedrops for at least 3 months prior to their initial visit. They were further divided into two subgroups according to the duration of diabetes (<15 vs. ≥15 years). All participants are among Han ethic group and are from eastern China. The exclusion criteria were patients with histories of lacrimal or blepharal diseases, corneal or conjunctival disorders, glaucoma, contact lens wearing, history of eye surgeries or with other systematic diseases other than T2DM.

Among these patients, T2DM and non-diabetic controls who were scheduled for cataract surgeries were further followed up twice: one after 3 days of topical application of 0.5% levofloxacin preoperatively (Cravit, Santen, Japan; three times a day), and the other at 7 days after ceasing all postoperative medications of cataract surgery, including 0.5% levofloxacin three times a day for 2 weeks, 1% Prednisolone acetate ophthalmic suspension (Pred Forte, Allergan, Ireland) three times a day for 2 weeks, and Diclofenac sodium eye drops (Difei, Sinqi, China) three times a day for 4 weeks.

### Sample Collection

A randomly chosen eye from each participant was sampled at their initial visit, which was defined as baseline status. The Lens Opacities Classification System III (LOCS III) scale was used to determine the severity of cataract for all participants. Each scale is decimalized ranging from 0.1 (a completely clear or colorless lens) to 6.9 (upper value on the nuclear color). Their prior chosen eye was sampled repeatedly during the two follow-ups. Samples were collected using disposable sterile dry absorbent cotton swabs without anesthesia, placed in sterile tubes and stored in a freezer at −80°C before DNA extraction (18). A single sample from each patient was obtained and the lower conjunctival fornix was scrubbed. The flow diagram of our study procedures was demonstrated in Supplementary Figure 1.

### DNA Extraction and Amplification

Following manufacturer's directions, DNA from all samples was extracted using MasterPure™ Complete DNA and RNA purification Kit (Epicenter, Madison, USA). The purity was assessed by NanoDrop 2000 Spectrophotometer, and the DNA integrity was verified by 0.8% agarose gel electrophoresis. Negative controls consisting of empty sterile storage tubes were processed for DNA extraction and no detectable amplification was observed.

The hypervariable region V3-V4 of the 16S rDNA gene were amplified by KAPA HiFi Hotstart ReadyMix PCR kit with barcoded primers (314F 5′-CCTACGGGRSGCAGCAG-3′; 806R 5′-GGACTACVVGGGTATCTAATC-3′). PCR reactions were performed in 30 μL mixture containing 15 μL of 2 × KAPA Library Amplification ReadyMix, 1 μL of each primer (10 μM), 50 ng of template DNA and ddH2O. Thermal cycling consisted of an initial denaturation at 95°C for 3 min, followed by 30 cycles at 98°C for 20 s, 58°C for 15s, and 72°C for 20s and a final extension at 72°C for 5 min. The PCR products were purified using a AxyPrep Gel Extraction Kit (Axygen Biosciences, Union City, CA, U.S.) and quantified using Qubit^®^2.0 (Invitrogen, U.S.). Amplicons with concentration ≥5 ng/μl and OD_260_/OD_280_ = 1.8–2.0 were considered qualified. All quantified amplicons were pooled to equalize concentrations for sequencing using Illumina MiSeq platform (Illumina, CA, USA). The paired end reads of 250 bp were overlapped on their three ends for concatenation into original longer tags by using PANDAseq (https://github.com/neufeld/pandaseq, version 2.9).

### 16S Sequencing and Data Analysis

Low-quality tags were filtered based on the following criteria: (1) with average Phred score of bases worse than 20; (2) with more than three ambiguous N bases; and (3) with length of <220 or >500 nt. Only the tags with frequency more than 1, which tend to be more reliable, were clustered into operational taxonomic units (OTUs) by UPARSE (http://drive5.com/uparse/) with a similarity threshold of 97% (19). Representative OTU sequences were taxonomically determined using naïve Bayesian Ribosomal Database Project (RDP) classifier against the RDP database (http://rdp.cme.msu.edu/) using confidence threshold of 0.8 (20). OTU profiling table and diversity analyses were also achieved by python scripts of QIIME (version 1.9.1) (21). The α-diversity was measured by observed species, Chao 1, Shannon, and Simpson indices. The β-diversity was measured by unweighted UniFrac demonstrating the difference or similarity between groups, and principle coordinates analysis (PCoA) plots were constructed to visually present the distance among samples. Linear discriminant (LDA) analysis effect size (LEfSe) analysis was used to further analyze the significance of the difference in the bacterial distribution at phylum and genus level (22), and LDA score >2.0 was set as the threshold. The Phylogenetic Investigation of Communities by Reconstruction of Unobserved States (PICRUSt, http://huttenhower.sph.harvard.edu/galaxy) analysis was used to predict metagenome functions with Kyoto Encyclopedia of Genes and Genomes (KEGG) database (23, 24).

### Availability of Data and Materials

The dataset supporting the conclusions of this article is available in the Sequence Read Archive of NCBI (https://www.ncbi.nlm.nih.gov/sra) under accession number PRJNA629667.

### Statistics

For demographic comparisons, the student's *t*-test was used to compare the continuous variables while the chi-squared test was used to compare categorical variables. The α-diversity and the relative abundance of taxa were compared using Wilcoxon signed ranks tests. For PCoA analysis, Adonis statistical method was used to compare the differences. Statistical analyses were carried out with QIIME and R 3.5.1 software. *P* < 0.05 was considered to be statistically significant.

## Results

### Demographics of Participants and Taxonomic Assignment of the Conjunctival Microbiome

Conjunctival swab samples from 79 T2DM patients and 113 non-diabetic controls were successfully amplified and sequenced reliable data, and among them, 21 T2DM patients and 14 non-diabetic controls were in the follow-up group. The general characteristics of the subjects were summarized in Table 1. The age, sex, and LOCS III gradings showed no differences between controls and T2DM patients in the baseline and the follow-up groups, or between the two T2DM subgroups with different durations.

**Table 1:** Demographics.

**Parameters**	**Baseline**		***P*-value**	**T2DM subgroup**		***P*-value**	**Follow-up group**		***P*-value**
	**Control**	**T2DM**		**T2DM <15 years**	**T2DM ≥15 years**		**Non-diabetic**	**T2DM**	
Number	113	79		50	29		14	21	
Age (mean ± SD)	65 ± 10	67 ± 8	>0.05	66 ± 9	69 ± 8	>0.05	66 ± 12	66 ± 10	>0.05
Sex (Female)	61	47	>0.05	28	18	>0.05	9	13	>0.05
LOCS III NC scale (mean ± SD)	2.6 ± 1.5	2.8 ± 1.4	>0.05	2.7 ± 1.2	2.8 ± 1.0	>0.05	3.0 ± 1.1	3.2 ± 1.4	>0.05

*T2DM, type 2 diabetes mellitus; LOCS, the Lens Opacities Classification System; NC, nuclear color; SD, standard deviation*.

*Student's t-test was used to compare the continuous variables while the Pearson's chi-squared test was used to compare categorical variables. Spearman correlation analysis was used for statistical comparisons of LOCS III scores*.

By 16S rDNA sequencing, we obtained 35,089 clean reads on average per sample (ranged from 29,133 to 38,977). Clean reads were mapped to 1,329 OTUs in total by RDP classifier. The mean number of OTUs per sample was 97 ± 53 (ranged from 9 to 272, Supplementary Table 1).

### Comparisons of Baseline Conjunctival Microbiome of the Controls and T2DM Patients

**Figure 1:**
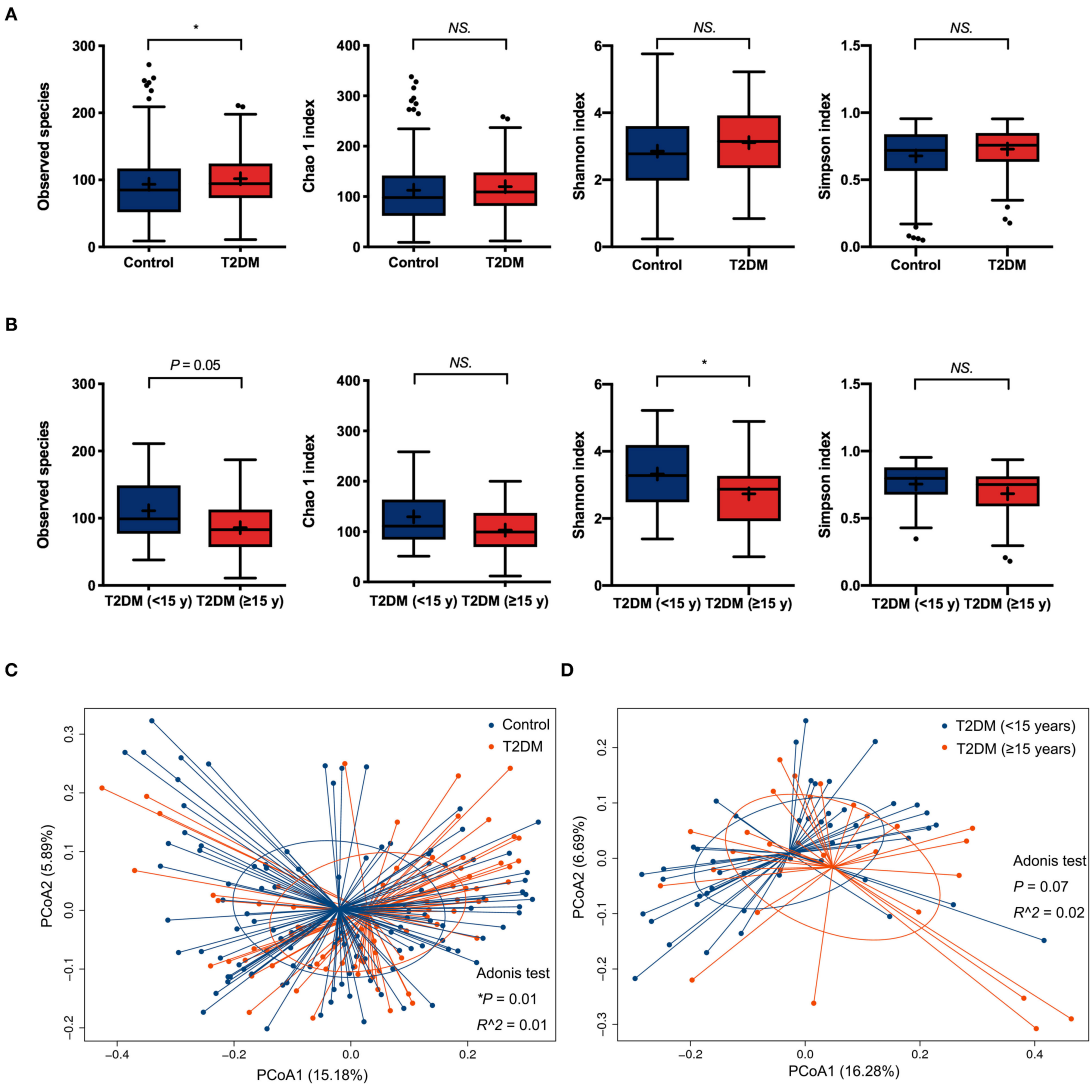
The α-diversity and β-diversity analysis of the conjunctival microbiota. **(A)** Comparisons of α-diversity indices between the controls and T2DM patients (113 vs. 79 samples). **(B)** Subgroups comparisons of α-diversity indices between patients with less and more than 15 years' duration of T2DM (50 vs. 29 samples). Wilcoxon signed-rank test was used to compare the difference. **(C)** Comparison of β-diversities between the controls and T2DM patients (113 vs. 79 samples). **(D)** Comparison of β-diversities between patients with less and more than 15 years' duration of T2DM (50 vs. 29 samples). Wilcoxon signed-rank test was used to compare the α-diversity difference, and Adonis test was used to compare the β-diversity difference between two groups. NS. means no significance; *P < 0.05.

For α-diversity analysis, as compared to non-diabetic controls, the observed species index was significantly higher in the conjunctival microbiome of T2DM patients (*P* < 0.05). The Chao 1, Shannon, and Simpson indices tended to be higher in the T2DM group as well, but without statistical significance (Figure 1A). T2DM subgroup comparisons revealed less microbial diversity in the conjunctival microbiome of patients with ≥15 years' duration, as indicated by marginally lower observed species (*P* = 0.05) and statistically lower Shannon index compared to patients with <15 years' duration (*P* < 0.05, Figure 1B). The β-diversity analysis revealed a significant difference in the bacterial composition of ocular surface microbiota between the controls and T2DM patients, demonstrated by PCoA plots based on unweighted UniFrac (Adonis *P* < 0.05, Figure 1C), while no statistical significance was found between the two T2DM subgroups (Figure 1D).

**Figure 2:**
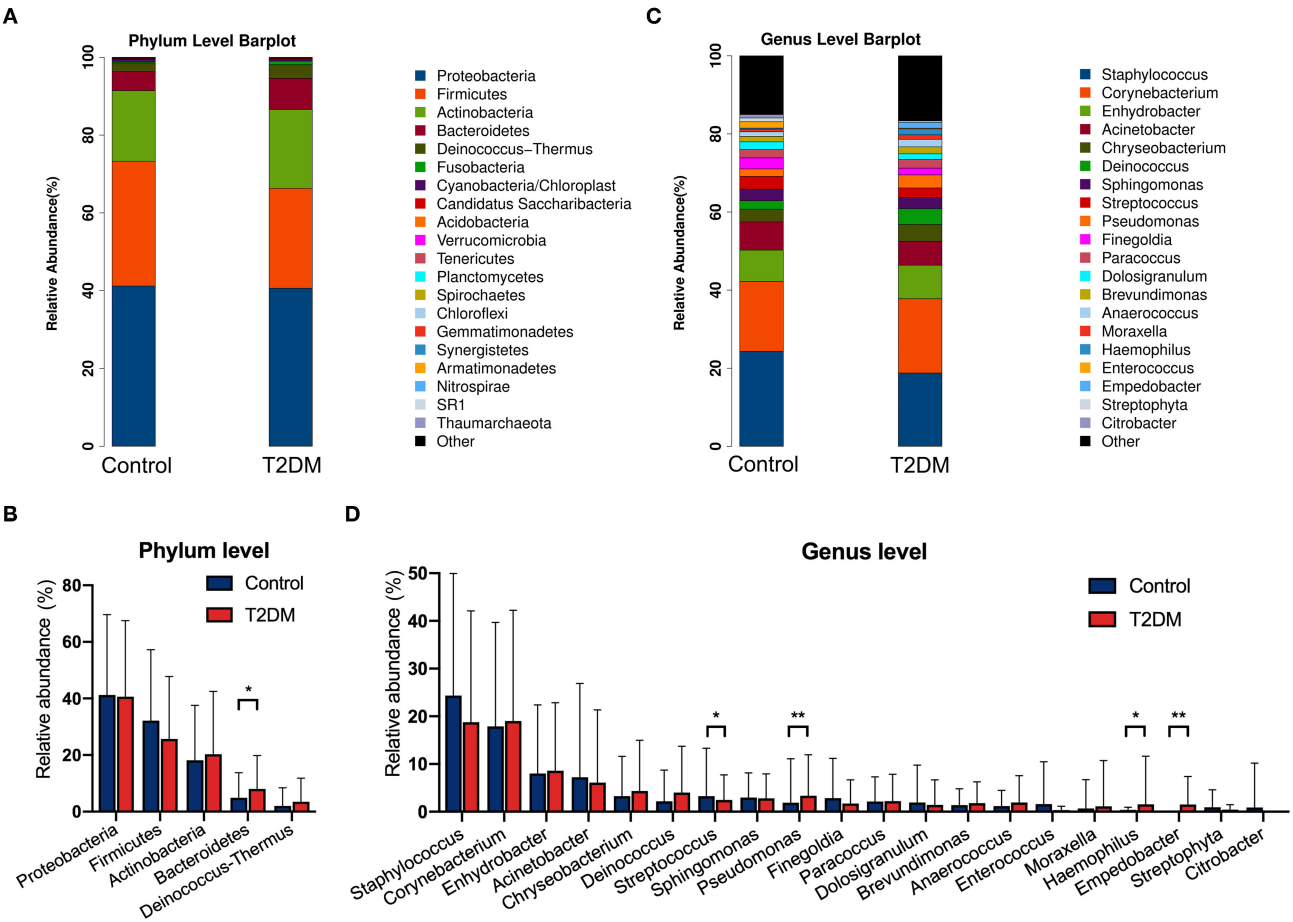
The relative abundances of conjunctival microbiota in the control group and in T2DM group. **(A)** The relative abundance at the phylum level. **(B)** Comparisons of the top 5 phyla between the control group and T2DM group (113 vs. 79 samples). **(C)** The relative abundance at the genus level. **(D)** Comparisons of the top 20 genera between the control group and T2DM group (113 vs. 79 samples). Wilcoxon signed-rank test was used. *P < 0.05; **P < 0.01.

**Figure 3:**
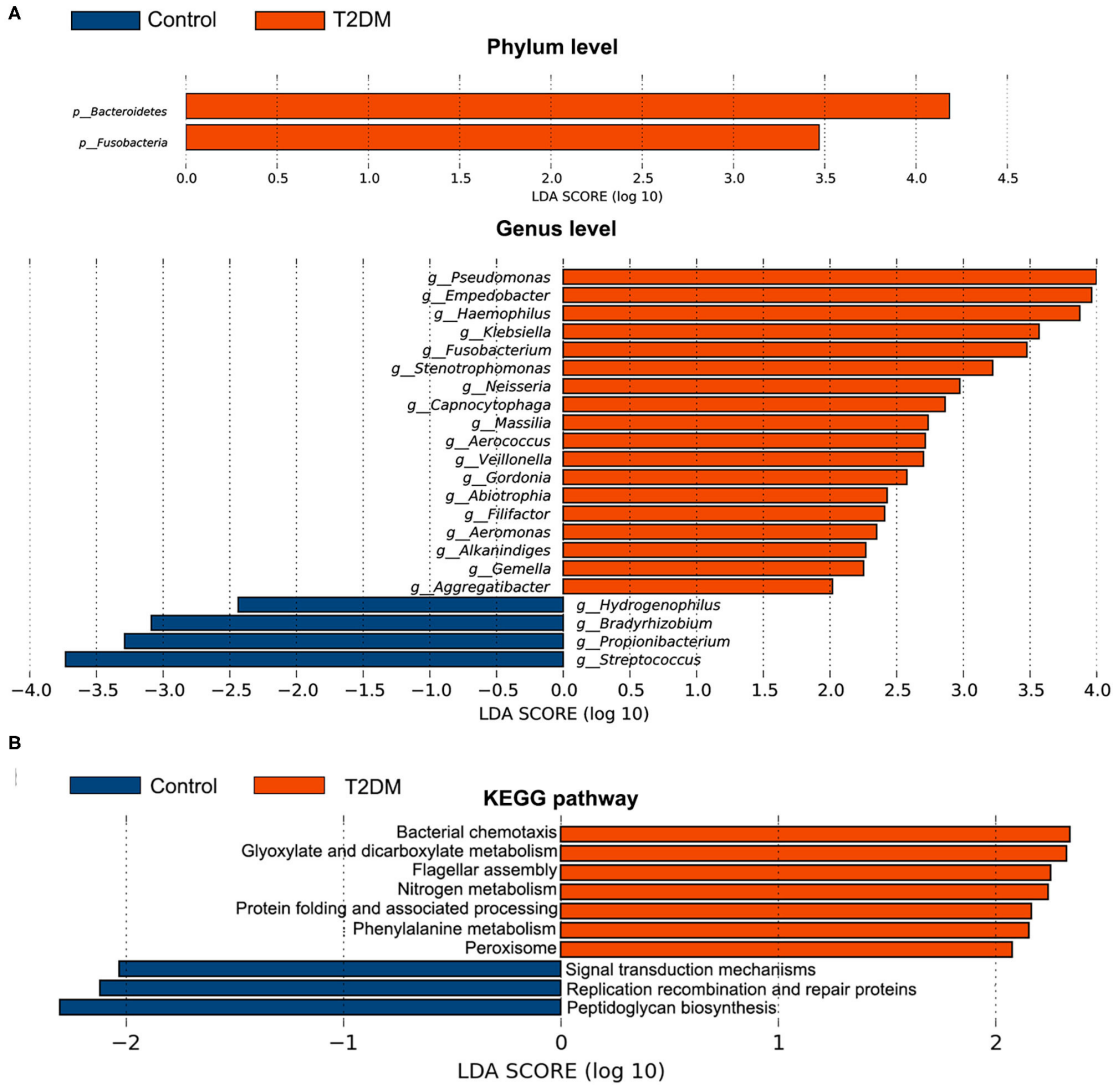
Comparative analysis of the conjunctival microbiota and the KEGG pathways between the control group and T2DM group. Bars represent linear discriminant analysis scores (LDA) based on LEfSe results. **(A)** The taxa with different relative abundance at phylum and genus levels. **(B)** The different KEGG pathways. Positive LDA score means enriched taxa or metabolic pathways in T2DM patients, while negative score means in the controls.

The top four most abundant phyla in conjunctival microbiota of both the controls and T2DM patients were *Proteobacteria* (41.25 and 40.65%), *Firmicutes* (32.14 and 25.73%), *Actinobacteria* (18.17 and 20.31%), and *Bacteroidetes* (4.89 and 8.02%, *P* < 0.05, Figures 2A,B). The top 20 most abundant genera were listed in Figure 2C, among which the genus *Pseudomonas, Haemophilus*, and *Empedobacter* were more enriched in the T2DM group, while the genus *Streptococcus* was more enriched in the control group (Figure 2D, *P* < 0.05). LEfSe analysis verified that phylum *Bacteroidetes* and *Fusobacteria*, as well as genus *Pseudomonas, Empedobacter, Haemophilus, Klebsiella, Fusobacterium, Stenotrophomonas, Neisseria, Capnocytophaga, Massilia, Aerococcus, Veillonella, Gordonia, Abiotrophia, Filifactor, Aeromonas, Alkanindiges, Gemella*, and *Aggregatibacter* were enriched on the ocular surface of T2DM patients, while the genus *Streptococcus, Propionibacterium, Bradyrhizobium*, and *Hydrogenophilus* were enriched on the ocular surface of the controls (Figure 3A). The PICRUSt analysis revealed significantly elevated metabolic pathways related to cell mobility, including bacterial chemotaxis and flagellar assembly. Other elevated pathways included glyoxylate and dicarboxylate metabolism, nitrogen metabolism and etc. in the conjunctival microbiome of T2DM patients as compared to that of the controls (Figure 3B).

### Alterations of Conjunctival Microbiome in Response to Topical Use of Levofloxacin

**Figure 4:**
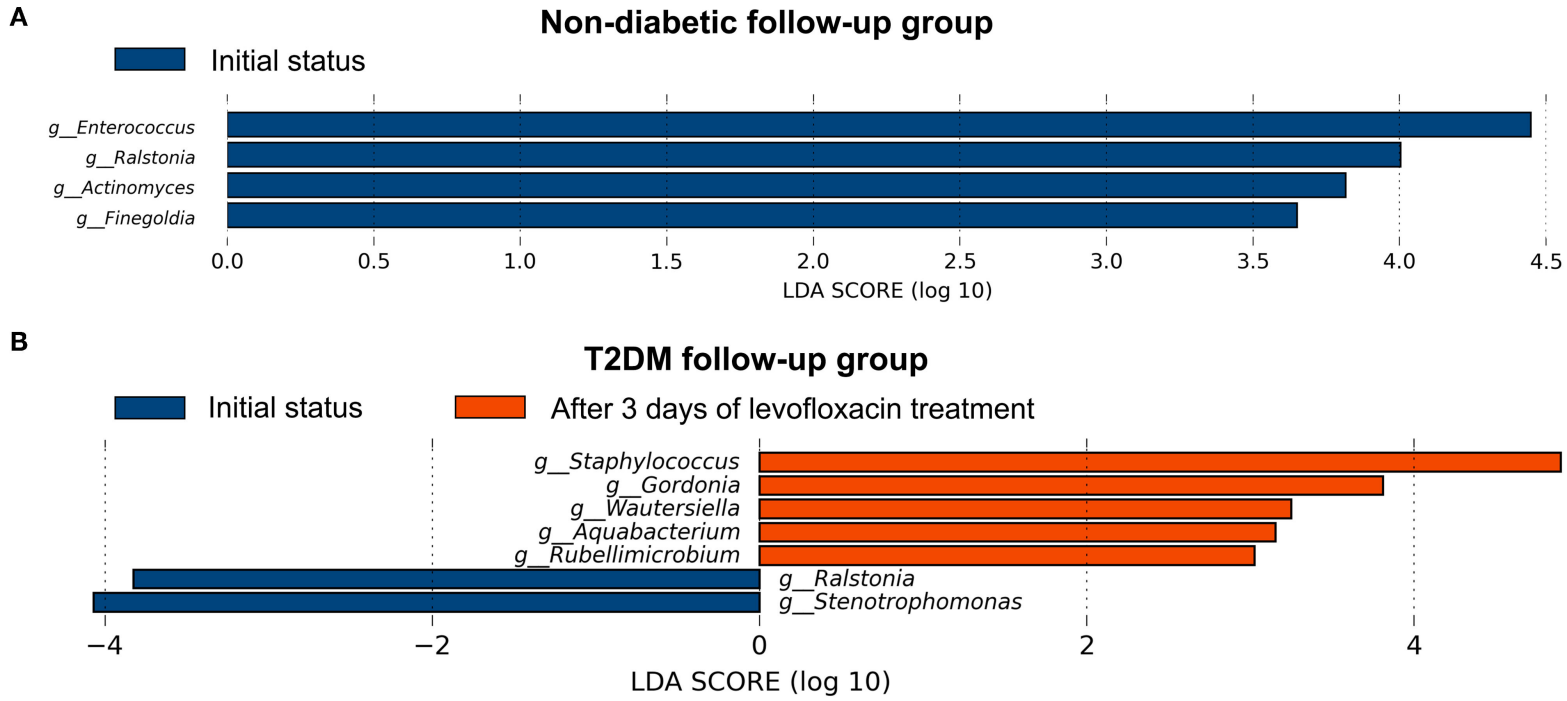
Distinct alternations of conjunctival microbiota in response to levofloxacin eye drops based on LEfSe analysis at genus level. The statistically different genus in the ocular surface of the non-diabetic **(A)** and T2DM follow-up groups **(B)**, respectively. Positive linear discriminant analysis (LDA) score means the genus was more enriched before than after levofloxacin, while negative score means the genus was more enriched after than before levofloxacin.

After preoperative use of levofloxacin topically for 3 days and before any surgical procedures, the α- and β-diversity of the conjunctival microbiome were found no significant difference in both follow-up groups compared to their initial status (Supplementary Table 2, Supplementary Figures 2A,B). After using levofloxacin, distinct alterations were revealed in the microbial composition of the two follow-up groups respectively. In the non-diabetic follow-up group, the genus *Enterococcus, Ralstonia, Actinomyces*, and *Finegoldia* were more enriched at initial status, while after levofloxacin treatment, no genus was observed more enriched in the conjunctival microbiota (Figure 4A). In the T2DM follow-up group, the genus *Stenotrophomonas* and *Ralstonia* were more enriched at initial status, while the genus *Staphylococcus, Gordonia, Wautersiella, Aquabacterium*, and *Rubellimicrobium* were more enriched after levofloxacin treatment (Figure 4B).

### The Resilience of Conjunctival Microbiome After Ceasing All Postoperative Medications of Cataract Surgery

We then longitudinally compared the conjunctival microbiome at 7 days after ceasing all postoperative medications of cataract surgery to their initial status in both diabetic and non-diabetic follow-up groups respectively. No significant difference was observed in α- or β-diversity of the conjunctival microbiome between these two time points of T2DM and non-diabetic follow-up groups (Supplementary Table 2, Supplementary Figures 2C,D). No taxa with significant enrichment or reduction was found neither in the comparative analysis. Our data suggested that the conjunctival microbiome might be restored to initial status at 7 days after ceasing all postoperative medications of cataract surgery.

## Discussion

Researches on ocular surface flora in diabetic patients started previously with culturing techniques, and higher bacterial isolation rates were revealed on the ocular surface of diabetic patients (6, 25). Nevertheless, traditional culturing methods had limitations in the integrity and throughput. As the development of NGS techniques, mapping the relatively more thorough microbiota of ocular surface became possible. There were a few studies with small sample sizes revealing the microbial diversities and compositions in diabetic conjunctiva using 16S rDNA sequencing (15, 16), yet studies with larger sample sizes are still rare. On the other hand, due to higher risk of ocular infections and complications after ocular surgical procedures in T2DM patients, the influence of perioperative topical antibiotics on the conjunctival microbiota was also noteworthy.

Diabetic patients are known to have an increased risk of infections (26, 27). Systemic and regional infections in these patients can be related to impaired immunity and characteristic distributions of gut (28, 29), skin (30), and subgingival (31) microbiome. Ocular infections were also found more frequent in diabetic patients (8, 32). Normally, the conjunctiva serves as physical barrier of the eye, and contributes to mucosal immunity (33). In patients with T2DM, the composition of core and transient conjunctival microbiota may have altered from non-diabetic subjects. Clarification of the distinct conjunctival microbiome in T2DM patients might provide potential explanations for the higher risk of ocular infections.

In this study, we firstly demonstrated the baseline differences of conjunctival microbiome in T2DM patients from the controls. The conjunctival microbiome in T2DM patients tended to be more diverse than in the controls as indicated by slightly higher Shannon and Simpson indices (though without statistical significance), which was also in concert with previous studies (15, 16). A similar trend was also reported in oral microbiome of diabetic mice (34). The microbial diversity seemed to be related to the duration of T2DM. Our data showed the conjunctival microbiome of T2DM patients with longer duration was less diverse, which might suggest the low-diversity dysbiosis on the ocular surface at the later stage of disease (35). Lower microbial diversity found in nasal microbiota was considered to be associated with higher risk of respiratory viral infections (36). Thus, lower α-diversity in the conjunctival microbiota of T2DM patients with >15 years' duration may also suggest a higher risk of ocular infection.

Despite the individual differences, the top four phyla in both groups were similar to previous findings of core microbiome on ocular surface (16, 18, 37, 38), and also the core microbiome found in conjunctival swabs by Ozkan et al. (39). The significant enrichment of phylum *Bacteroidetes* was revealed in the conjunctival microbiome of T2DM patients, which was in concert with previous reports in diabetic conjunctival (15) or gut microbiome (29). At genus level, it seems that the differences were mainly among the lower-abundance genera. Among the top 20 genera, our data also showed the enrichment of genus *Pseudomonas, Haemophilus*, and *Empedobacter* on the ocular surface of T2DM patients. Some of the species under these genera were pathogenic causing ocular surface infections. For example, *Pseudomonas aeruginosa* was commonly isolated in ocular infections (40), and *Haemophilus influenzae* was considered the leading cause of conjunctivitis (14). The low-abundance bacteria might be more susceptible to the alternation of the microenvironment in the disease status, especially for the systematic diseases due to the potential and subtle influence, instead of drastic and direct influence generated from ocular diseases.

As for the microbial genes functioning, our results showed pathways related to cell motility including the bacterial chemotaxis and the flagellar assembly were significantly enriched on the ocular surface of T2DM patients. The bacterial chemotaxis is the motility in response to adverse conditions, which is a critical ability to search for the optimal environment to ensure the survival of bacterial species. The elevated cell mobility might enable the bacteria to modulate their swimming behavior, which was considered to be related to bacterial inflammatory processes (41) and plays an important role in the onset of infections (42). Furthermore, nitrogen metabolism was also elevated (including many transporters, enzymes, regulators, etc.), which was essential for the full virulence of pathogens (43). These increased bacterial metabolic pathways might because of the hyperglycemia and impaired innate immunity of T2DM patients. Thus, our data suggested a more susceptible status for infections of the conjunctival microbiome in T2DM patients.

Accordingly, knowing the influence of topical antibiotics on the conjunctival microbiome of T2DM patients was important. Based on our longitudinal comparisons, no significantly enriched genus was found after levofloxacin treatment compared with the initial status in the conjunctival microbiome of the controls. However, in that of the T2DM patients, five genera were found noticeably more abundant afterwards, including the genus *Staphylococcus*. Meanwhile, a previous study reported the development of tolerance to levofloxacin of *Staphylococcus epidermidis* isolated from ocular surface after ceasing topical antibiotics after cataract surgery (44). Hence, though the bacterial diversity was not influenced by the use of antibiotics, the preoperative treatment with levofloxacin might somehow increase the abundance of potential pathogens in T2DM patients.

Although post-cataract-surgery endophthalmitis became very rare with intraoperative injections of antibiotics, the risk was still much higher in patients with diabetes than in those without (OR = 2.92, 95% CI: 1.72–4.96) (9). The immune dysfunction with the increased bacterial diversity and cell mobility in T2DM patients might somehow be associated with a higher risk of ocular infections after ocular surgery, including exogenous endophthalmitis.

The resilience of conjunctival microbiota after cataract surgery is also important. The long-term effect of cataract surgery on ocular bacterial flora was previously revealed by culturing and isolation methods, and the positive isolation rate at their first postoperative sampling timepoint (3 months after ceasing application of antibiotics) showed no difference from the preoperative status (44). According to our data, the diversity and distribution of the conjunctival microbiome can be restored 7 days after ceasing all postoperative medications regardless of the T2DM presence, indicating the influence of surgical procedures and perioperative topical antibiotics might be eliminated within a short period. Further investigation and validation on how the microbiota is restored might require more frequent and continuous samplings and observations.

In conclusion, the conjunctival bacterial microbiome in T2DM patients was more complex than that in non-diabetic controls. The ocular surface microbiome of T2DM patients might present less sensitivity to perioperative use of topical antibiotics, and might be restored to the initial status in a short period after cataract surgery. Our data could provide potential clues to the higher risks of ocular infections and endophthalmitis after ocular surgical procedures among patients with T2DM.

## Data Availability Statement

The datasets presented in this study can be found in online repositories. The names of the repository/repositories and accession number(s) can be found at: (https://www.ncbi.nlm.nih.gov/sra), PRJNA629667.

## Ethics Statement

The studies involving human participants were reviewed and approved by Ethics Committee of Eye and Ear, Nose, and Throat Hospital of Fudan University. The patients/participants provided their written informed consent to participate in this study.

## Author Contributions

XZ and LiW researched data and wrote the manuscript. XR collected samples and researched data. XZ, FC, YL, and LaW reviewed and edited the manuscript. YL, QZ, and XW contributed to the discussion and edited the manuscript. WH and KZ contributed to sample collecting. All authors contributed to the article and approved the submitted version.

## Conflict of Interest

The authors declare that the research was conducted in the absence of any commercial or financial relationships that could be construed as a potential conflict of interest.

## Abbreviations

T2DM: type 2 diabetes mellitus
NGS: next-generation sequencing
LOCS III: The Lens Opacities Classification System III
OTUs: operational taxonomic units
RDP: Ribosomal Database Project
LDA: Linear discriminant
LEfSe: Linear discriminant analysis effect size
KEGG: Kyoto Encyclopedia of Genes and Genomes
PICRUSt: Phylogenetic Investigation of Communities by Reconstruction of Unobserved States
PCoA: principle coordinates analysis.

## References

[1] International Diabetes Federation. IDF Diabetes Atlas. (2019). Avilable online at: http://www.diabetesatlas.org/ (accessed January 23, 2020).

[2] Tao Z Shi A Zhao J. Epidemiological perspectives of diabetes. Cell Biochem Biophys. (2015) 73:181–5. 10.1007/s12013-015-0598-4 25711186

[3] Abu-Ashour W Twells LK Valcour JE Gamble JM. Diabetes and the occurrence of infection in primary care: a matched cohort study. BMC Infect Dis. (2018) 18:67. 10.1186/s12879-018-2975-2 29402218 PMC5800043

[4] Thimmappaiah Jagadeesh A Prakash PY Karthik Rao N Ramya V. Culture characterization of the skin microbiome in type 2 diabetes mellitus: a focus on the role of innate immunity. Diabetes Res Clin Pract. (2017) 134:1–7. 10.1016/j.diabres.2017.09.007 28951341

[5] Kawata T Matsuo T. Positive bacterial culture in conjunctival sac before cataract surgery with night stay is related to diabetes mellitus. BMC Ophthalmol. (2017) 17:14. 10.1186/s12886-017-0413-7 28219351 PMC5319027

[6] Adam M Balci M Bayhan HA Inkaya AC Uyar M Gurdal C. Conjunctival flora in diabetic and nondiabetic individuals. Turk J Ophthalmol. (2015) 45:193–6. 10.4274/tjo.33230 27800231 PMC5082240

[7] Negi A Vernon SA. An overview of the eye in diabetes. J R Soc Med. (2003) 96:266–72. 10.1258/jrsm.96.6.266 12782689 PMC539505

[8] Ansari AS de Lusignan S Hinton W Munro N McGovern A. The association between diabetes, level of glycaemic control and eye infection: Cohort database study. Prim Care Diabetes. (2017) 11:421–9. 10.1016/j.pcd.2017.05.009 28648963

[9] Pershing S Lum F Hsu S Kelly S Chiang MF Rich WL 3rd. Endophthalmitis after cataract surgery in the United States: a report from the intelligent research in sight registry, 2013-2017. Ophthalmology. (2020) 127:151–8. 10.1016/j.ophtha.2019.08.026 31611015 PMC12313552

[10] Kattan HM Flynn HW Jr Pflugfelder SC Robertson C Forster RK. Nosocomial endophthalmitis survey. Current incidence of infection after intraocular surgery. Ophthalmology. (1991) 98:227–38. 10.1016/S0161-6420(91)32312-1 2008282

[11] Doft BH Wisniewski SR Kelsey SF Groer-Fitzgerald S. Diabetes and postcataract extraction endophthalmitis. Curr Opin Ophthalmol. (2002) 13:147–51. 10.1097/00055735-200206000-00003 12011682

[12] Lu LJ Liu J. Human microbiota and ophthalmic disease. Yale J Biol Med. (2016) 89:325−30. 27698616 PMC5045141

[13] Cavuoto KM Banerjee S Galor A. Relationship between the microbiome and ocular health. Ocul Surf. (2019) 17:384–92. 10.1016/j.jtos.2019.05.006 31125783

[14] Teweldemedhin M Gebreyesus H Atsbaha AH Asgedom SW Saravanan M. Bacterial profile of ocular infections: a systematic review. BMC Ophthalmol. (2017) 17:212. 10.1186/s12886-017-0612-2 29178851 PMC5702129

[15] Li S Yi G Peng H Li Z Chen S Zhong H. How ocular surface microbiota debuts in type 2 diabetes mellitus. Front Cell Infect Microbiol. (2019) 9:202. 10.3389/fcimb.2019.00202 31263683 PMC6590198

[16] Ham B Hwang HB Jung SH Chang S Kang KD Kwon MJ. Distribution and diversity of ocular microbial communities in diabetic patients compared with healthy subjects. Curr Eye Res. (2018) 43:314–24. 10.1080/02713683.2017.1406528 29172724

[17] Gower EW Lindsley K Tulenko SE Nanji AA Leyngold I McDonnell PJ. Perioperative antibiotics for prevention of acute endophthalmitis after cataract surgery. Cochrane Database Syst Rev. (2017) 2:CD006364. 10.1002/14651858.CD006364.pub3 28192644 PMC5375161

[18] Huang Y Yang B Li W. Defining the normal core microbiome of conjunctival microbial communities. Clin Microbiol Infect. (2016) 22:643 e647–12. 10.1016/j.cmi.2016.04.008 27102141

[19] Edgar RC. UPARSE: highly accurate OTU sequences from microbial amplicon reads. Nat Methods. (2013) 10:996–8. 10.1038/nmeth.2604 23955772

[20] Cole JR Wang Q Fish JA Chai B McGarrell DM Sun Y. Ribosomal database project: data and tools for high throughput rRNA analysis. Nucleic Acids Res. (2014) 42:D633–42. 10.1093/nar/gkt1244 24288368 PMC3965039

[21] Caporaso JG Kuczynski J Stombaugh J Bittinger K Bushman FD Costello EK. QIIME allows analysis of high-throughput community sequencing data. Nat Methods. (2010) 7:335–6. 10.1038/nmeth.f.303 20383131 PMC3156573

[22] Segata N Izard J Waldron L Gevers D Miropolsky L Garrett WS. Metagenomic biomarker discovery and explanation. Genome Biol. (2011) 12:R60. 10.1186/gb-2011-12-6-r60 21702898 PMC3218848

[23] Langille MG Zaneveld J Caporaso JG McDonald D Knights D Reyes JA. Predictive functional profiling of microbial communities using 16S rRNA marker gene sequences. Nat Biotechnol. (2013) 31:814–21. 10.1038/nbt.2676 23975157 PMC3819121

[24] Sanchez-Alcoholado L Castellano-Castillo D Jordán-Martínez L Moreno-Indias I Cardila-Cruz P Elena D. Role of gut microbiota on cardio-metabolic parameters and immunity in coronary artery disease patients with and without type-2 diabetes mellitus. Front Microbiol. (2017) 8:1936. 10.3389/fmicb.2017.01936 29051757 PMC5633746

[25] Martins EN Alvarenga LS Höfling-Lima AL Freitas D Zorat-Yu MC Farah ME. Aerobic bacterial conjunctival flora in diabetic patients. Cornea. (2004) 23:136–42. 10.1097/00003226-200403000-00006 15075882

[26] Frydrych LM Bian G O'Lone DE Ward PA Delano MJ. Obesity and type 2 diabetes mellitus drive immune dysfunction, infection development, and sepsis mortality. J Leukoc Biol. (2018) 104:525–34. 10.1002/JLB.5VMR0118-021RR 30066958

[27] Rayfield EJ Ault MJ Keusch GT Brothers MJ Nechemias C Smith H. Infection and diabetes: the case for glucose control. Am J Med. (1982) 72:439–50. 10.1016/0002-9343(82)90511-3 7036735

[28] Lambeth SM Carson T Lowe J Ramaraj T Leff JW Luo L. Composition, diversity and abundance of gut microbiome in prediabetes and type 2 diabetes. J Diabetes Obes. (2015) 2:1–7. 10.15436/2376-0949.15.031 26756039 PMC4705851

[29] Sohail MU Althani A Anwar H Rizzi R Marei HE. Role of the gastrointestinal tract microbiome in the pathophysiology of diabetes mellitus. J Diabetes Res. (2017) 2017:9631435. 10.1155/2017/9631435 29082264 PMC5634576

[30] Gardiner M Vicaretti M Sparks J Bansal S Bush S Liu M. A longitudinal study of the diabetic skin and wound microbiome. PeerJ. (2017) 5:e3543. 10.7717/peerj.3543 28740749 PMC5522608

[31] Zhou M Rong R Munro D Zhu C Gao X Zhang Q. Investigation of the effect of type 2 diabetes mellitus on subgingival plaque microbiota by high-throughput 16S rDNA pyrosequencing. PLoS ONE. (2013) 8:e61516. 10.1371/journal.pone.0061516 23613868 PMC3632544

[32] Kruse A Thomsen RW Hundborg HH Knudsen LL Sorensen HT Schonheyder HC. Diabetes and risk of acute infectious conjunctivitis–a population-based case-control study. Diabet Med. (2006) 23:393–7. 10.1111/j.1464-5491.2006.01812.x 16620267

[33] Knop E Knop N. Anatomy and immunology of the ocular surface. Chem Immunol Allergy. (2007) 92:36–49. 10.1159/000099252 17264481

[34] Xiao E Mattos M Vieira GHA Chen S Corrêa JD Wu Y. Diabetes enhances IL-17 expression and alters the oral microbiome to increase its pathogenicity. Cell Host Microbe. (2017) 22:120–8.e124. 10.1016/j.chom.2017.06.014 28704648 PMC5701758

[35] Kriss M Hazleton KZ Nusbacher NM Martin CG Lozupone CA. Low diversity gut microbiota dysbiosis: drivers, functional implications and recovery. Curr Opin Microbiol. (2018) 44:34–40. 10.1016/j.mib.2018.07.003 30036705 PMC6435260

[36] Korten I Mika M Klenja S Kieninger E Mack I Barbani MT. Interactions of respiratory viruses and the nasal microbiota during the first year of life in healthy infants. mSphere. (2016) 1:e00312–16. 10.1128/mSphere.00312-16 27904883 PMC5120172

[37] Lee SH Oh DH Jung JY Kim JC Jeon CO. Comparative ocular microbial communities in humans with and without blepharitis. Invest Ophthalmol Vis Sci. (2012) 53:5585–93. 10.1167/iovs.12-9922 22836761

[38] Zhou Y Holland MJ Makalo P Joof H Roberts CH Mabey DC. The conjunctival microbiome in health and trachomatous disease: a case control study. Genome Med. (2014) 6:99. 10.1186/s13073-014-0099-x 25484919 PMC4256740

[39] Ozkan J Willcox M Wemheuer B Wilcsek G Coroneo M Thomas T. Biogeography of the human ocular microbiota. Ocul Surf . (2019) 17:111–8. 10.1016/j.jtos.2018.11.005 30445178

[40] Michael KB Rotchford A Ramaesh K. Conjunctival chemosis as a specific feature of *Pseudomonas aeruginosa* corneal ulcers. Cornea. (2016) 35:1182–4. 10.1097/ICO.0000000000000947 27429077

[41] Zhang L Liu Y Zheng HJ Zhang CP. The oral microbiota may have influence on oral cancer. Front Cell Infect Microbiol. (2019) 9:476. 10.3389/fcimb.2019.00476 32010645 PMC6974454

[42] Kang Y Zhang H Hu M Ma Y Chen P Zhao Z. Alterations in the ocular surface microbiome in traumatic corneal ulcer patients. Invest Ophthalmol Vis Sci. (2020) 61:35. 10.1167/iovs.61.6.35 32543662 PMC7415308

[43] Yeung AT Janot L Pena OM Neidig A Kukavica-Ibrulj I Hilchie A. Requirement of the *Pseudomonas aeruginosa* CbrA sensor kinase for full virulence in a murine acute lung infection model. Infect Immun. (2014) 82:1256–67. 10.1128/IAI.01527-13 24379284 PMC3957987

[44] Ono T Nejima R Iwasaki T Mori Y Noguchi Y Yagi A. Long-term effects of cataract surgery with topical levofloxacin on ocular bacterial flora. J Cataract Refract Surg. (2017) 43:1129–34. 10.1016/j.jcrs.2017.06.037 28991607

