# Supplementary material for: Conjunctival Microbiota in Patients With Type 2 Diabetes Mellitus and Influences of Perioperative Use of Topical Levofloxacin in Ocular Surgery
Source: Front Med (Lausanne). 2021 Apr 6;8:605639. doi: 10.3389/fmed.2021.605639 (PMC8055849; doi:10.3389/fmed.2021.605639)
Supplement: Supplementary file 1 [file Data_Sheet_1.PDF]

**Supplementary Tab S1.** Taxonomic assignment.

| **Reads and OTUs** | **Statistics** |
|---|---|
| Clean reads | |
| Average | 35,089 |
| Range | (29,133, 38,977) |
| Mapped reads | |
| Average | 26,578 |
| Range | (10,417, 34,673) |
| Mapped ratio (%) | |
| Average | 75.68% |
| Range | (34.64%, 93.91%) |
| Number of OTUs | 1,329 |
| Assigned to Kingdom | 1,329 |
| Assigned to Phylum | 1,273 |
| Assigned to Class | 1,201 |
| Assigned to Order | 1,076 |
| Assigned to Family | 998 |
| Assigned to Genus | 895 |
| Assigned to Species | 0 |
| Mean ± SD (per sample) | 97 ± 53 |
| Range | (9, 272) |

OTU, operational taxonomic units, SD, standard deviation.
