# Supplementary material for: Conjunctival Microbiota in Patients With Type 2 Diabetes Mellitus and Influences of Perioperative Use of Topical Levofloxacin in Ocular Surgery
Source: Front Med (Lausanne). 2021 Apr 6;8:605639. doi: 10.3389/fmed.2021.605639 (PMC8055849; doi:10.3389/fmed.2021.605639)
Supplement: Supplementary file 2 [file Data_Sheet_2.PDF]

Supplementary Tab S2. The α-diversity indices compared longitudinally between the initial status and after 3 days of levofloxacin treatment or 7 days after ceasing all postoperative medications of cataract surgery in controls and T2DM patients.

**α-diversity Index**	**Initial status**	**After 3 days of levofloxacin treatment**	***P* value**	**7 days after ceasing all medications**	***P* value**
**Non-diabetic follow-up group**					
Observed species	42.57	35.23	0.29	45.50	0.56
Chao 1	51.98	48.12	0.56	55.39	0.62
Shannon	2.98	2.40	0.26	3.09	0.49
Simpson	0.72	0.60	0.26	0.71	0.66
**T2DM follow-up group**					
Observed species	45.00	56.18	0.25	42.70	0.93
Chao 1	52.28	65.40	0.18	45.59	0.61
Shannon	3.16	2.82	0.27	2.64	0.27
Simpson	0.75	0.67	0.35	0.67	0.44

Differences between the initial status and after 3 days of levofloxacin treatment or 7 days after ceasing all medications were compared using Wilcoxon signed-rank tests.
