# Supplementary figures and images for: Conjunctival Microbiota in Patients With Type 2 Diabetes Mellitus and Influences of Perioperative Use of Topical Levofloxacin in Ocular Surgery
Source: Front Med (Lausanne). 2021 Apr 6;8:605639. doi: 10.3389/fmed.2021.605639 (PMC8055849; doi:10.3389/fmed.2021.605639)

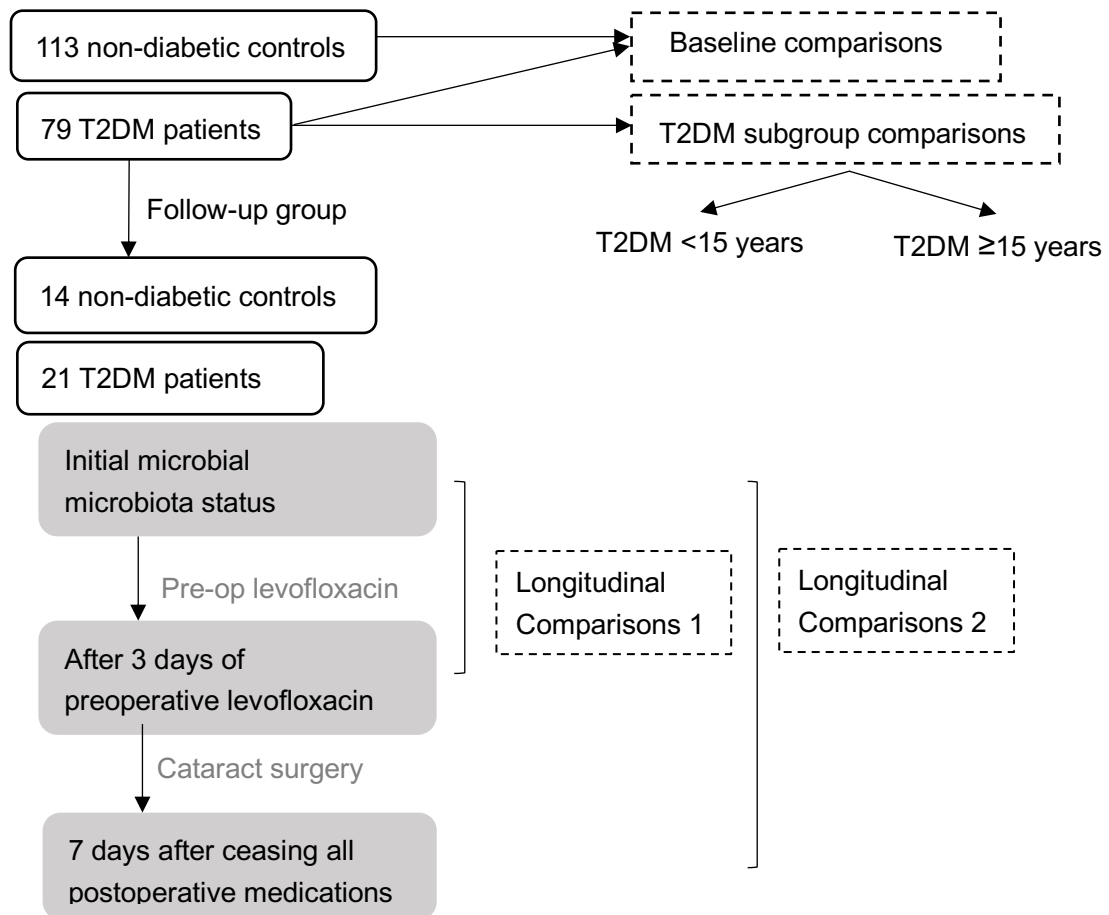

**Supplementary Figure S1.** The flow diagram of the study.

Supplement: Supplementary file 3 [file Image_1.pdf]
