# Supplementary material for: Conjunctival Microbiota in Patients With Type 2 Diabetes Mellitus and Influences of Perioperative Use of Topical Levofloxacin in Ocular Surgery
Source: Front Med (Lausanne). 2021 Apr 6;8:605639. doi: 10.3389/fmed.2021.605639 (PMC8055849; doi:10.3389/fmed.2021.605639)
Supplement: Supplementary file 4 [file Image_2.pdf]

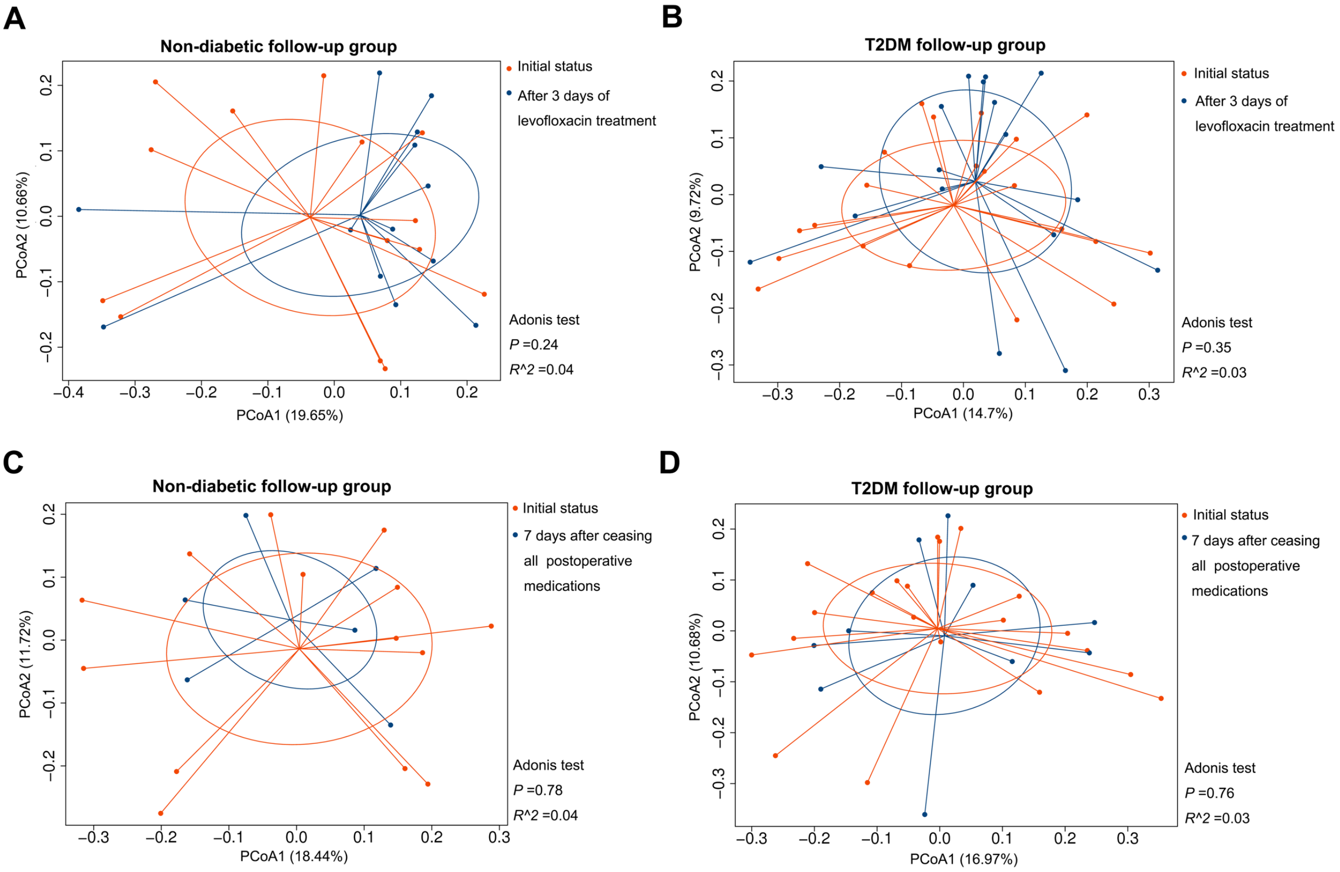

Supplementary Figure S2. The β-diversity compared by principle coordinate analysis (PCoA) plots based on unweighted Unifrac of the conjunctival microbiota between the initial status and after 3 days of levofloxacin in the controls (A) or T2DM group (B), and between the initial status and 7 days after ceasing all postoperative medications of cataract surgery in control (C) or T2DM group (D). Adonis test was used to compare the differences.
